# Automated ChIPmentation procedure on limited biological material of the human blood fluke *Schistosoma mansoni*

**DOI:** 10.12688/wellcomeopenres.17779.1

**Published:** 2022-04-11

**Authors:** Chrystelle Lasica, Ronaldo de Carvalho Augusto, Hélène Moné, Gabriel Mouahid, Cristian Chaparro, Anne-Clémence Veillard, Agnieszka Zelisko-Schmidt, Christoph Grunau

**Affiliations:** 1 IHPE, Univ Perpignan Via Domitia, CNRS, IFREMER, Univ Montpellier, Perpignan, 66860, France; 2 LBMC, Laboratoire de Biologie et Modélisation de la Cellule Univ Lyon, ENS de Lyon, Université Claude Bernard Lyon 1, CNRS, UMR 5239, INSERM, U1210,, Lyon, 69000, France; 3 Diagenode, Seraing, 4100, Belgium

**Keywords:** ChIPmentation, ChIP-seq, N-ChIP, limited biological material, Epigenetics, Schistosoma mansoni

## Abstract

In living cells, the genetic information stored in the DNA sequence is always associated with chromosomal and extra-chromosomal epigenetic information. Chromatin is formed by the DNA and associated proteins, in particular histones. Covalent histone modifications are important bearers of epigenetic information and as such have been increasingly studied since about the year 2000. One of the principal techniques to gather information about the association between DNA and modified histones is chromatin immunoprecipitation (ChIP), also combined with massive sequencing (ChIP-Seq). Automated ChIPmentation procedure is a convenient alternative to native chromatin immunoprecipitation (N-ChIP). It is now routinely used for ChIP-Seq in many model species, using in general roughly 10 ^6^ cells per experiment. Such high cell numbers are sometimes difficult to produce. Using the human parasite *Schistosoma mansoni*, whose production requires sacrificing animals and should therefore be kept to a minimum, we show here that automated ChIPmentation is suitable for limited biological material. We define the operational limit as ≥20,000 *Schistosoma* cells. We also present a streamlined protocol for the preparation of ChIP input libraries.

## Introduction

**Figure 1.**
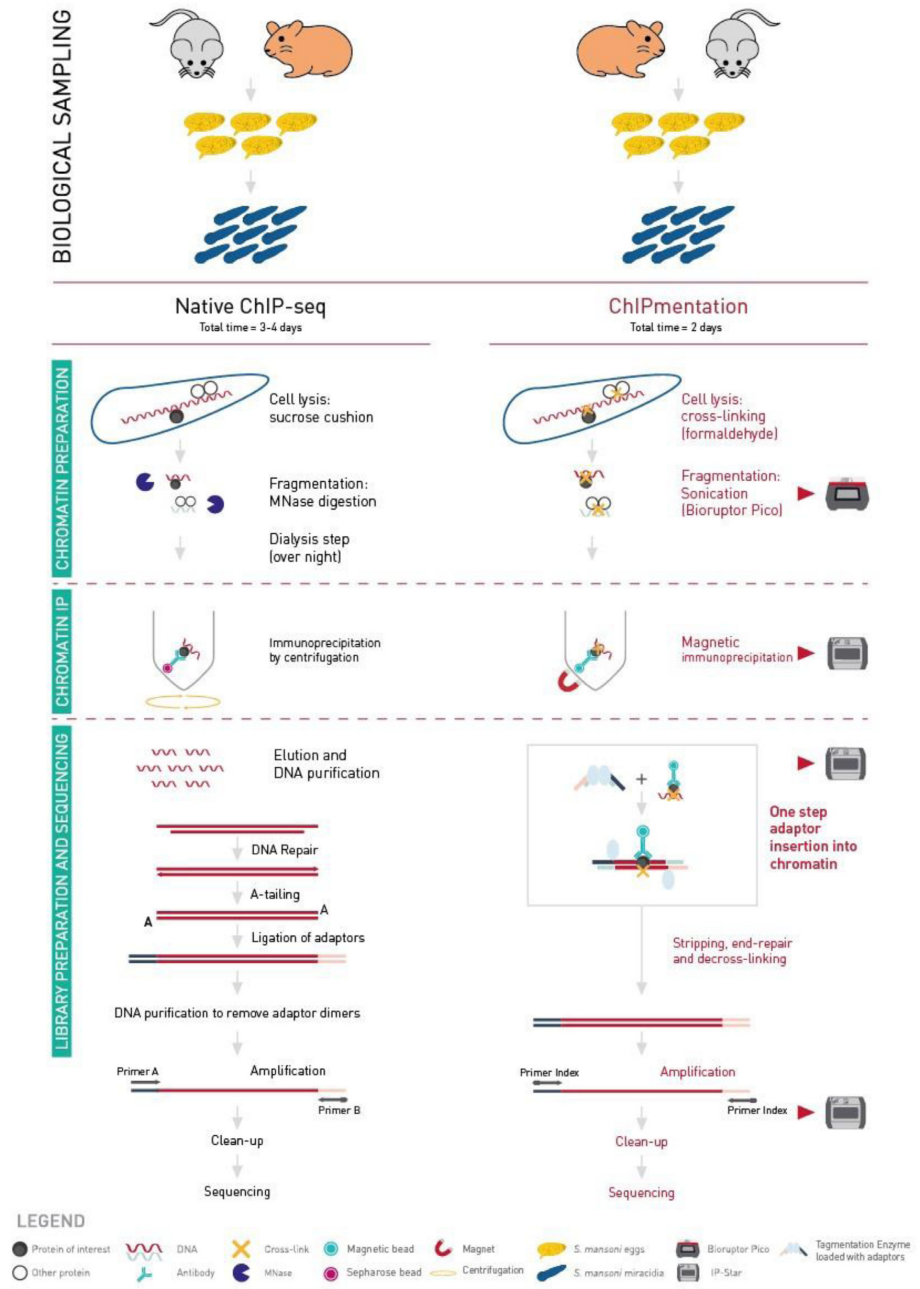
Comparison of Native-ChIP and ChIPmentation workflows. Biological sampling time depends on the biological model used. Native-ChIP protocol lasts three to four days. A sucrose cushion is used for cell lysis and MNase digestion for the fragmentation step. The immunoprecipitation is done by centrifugation. The whole process is done manually. ChIPmentation protocol lasts two days. Cross-linking is used for cell lysis and sonication for the fragmentation step. The immunoprecipitation, tagmentation and library cleaning are done with the IP-Star.

*Schistosoma mansoni* is a human parasite with a complex life cycle that shows strong developmental phenotypic plasticity, with intra-molluscal and intra-vertebrate stages, and two free-swimming larvae stages (miracidium and cercariae). We had shown by native chromatin immunoprecipitation (N-ChIP) that the different life cycle stages also show strong histone modification plasticity ( Augusto *et al.,* 2019; Cosseau *et al.,* 2009; Roquis *et al.,* 2018). While N-ChIP has been successfully used, we found that it is associated two challenges: one is the high hands-on time with the N-ChIP, and the other is obtaining enough biological material to perform several ChIP experiments with different antibodies. We therefore explored and benchmarked an automated ChIP procedure ( Figure 1). ChIPmentation is a ChIP-sequencing (ChIP-seq) technology which uses a transposase to add the sequencing adaptors to the DNA of interest instead of the classical multi-step processing, including end repair, A-tailing, adaptor ligation and size-selection ( Schmidl *et al.,* 2015). Thanks to the action of the transposase, loaded with sequencing adaptors, the library preparation is performed in only one step, which reduces hands-on time and material loss. Moreover, in the ChIPmentation approach, this tagmentation process is performed directly on chromatin during the immunoprecipitation process instead of naked DNA after purification. This workflow allows for a more reproducible tagmentation.

The combined facts that ChIPmentation has been automated on Diagenode’s IP-Star Compact Automated System and that this technology has been validated on low amounts of human cells ( Roels *et al.,* 2020; Schmidl *et al.,* 2015) make it a perfect candidate for ChIP-seq on limited material of other, non-model species. Here we addressed the questions of whether ChIPmentation, which was originally developed for human cell cultures, (i) can be used with schistosomes, (ii) whether it can be automated on a pipetting robot, and finally (iii) what the lowest schistosome cell number would be to obtain robust results with this procedure. We show here that the method is almost as sensitive as N-ChIP, but is about two times faster and can be carried out on the IP-Star pipetting robot, reducing experimenter hands-on and, more importantly, training time.

## Methods

### Production of biological material

*S. mansoni* NMRI eggs were extracted from livers of golden hamsters (donated by ParaDev) 42 days post-infection. Nocturnal *S. mansoni* from Oman ( Mouahid *et al.,* 2012) were extracted from livers of two swiss OF1 mice eight months post-infection. Miracidia were allowed to hatch for two hours in spring water, collected by pipetting and sedimented on ice for 30 min. Miracidia were counted under a microscope, aliquoted and stored at -80°C.

### Ethical considerations

Experiments on animals permits The laboratory received the permit number A 66040 for experiments on animals, from both the French *Ministère de l’Agriculture et de la Pêche* (Ministry of Agriculture and Fisheries) and the French *Ministère de l’Education Nationale de la Recherche et de la Technologie* (Ministry of Education, Research and Technology, Décret n° 2001-464 du 29 mai 2001 modifiant le décret n° 87-848 du 19 octobre 1987). This includes housing, breeding and care of the mice and hamsters, and animal experimentation. HM holds the official certificate for animal experimentation N° C661101 delivered by the *Direction Départementale de la Protection des Populations* (Articles R 214-87 à R 214-122 du Code Rural et article R 215-10 ; Arrêté du 19 avril 1988).

Ethical approval number: we obtained approval from the CEEA - 036 *Comité d’éthique en expérimentation animale Languedoc Roussillon* (CEEA-LR)”, which is the registration code of our ethical committee within the *Comité national de réflexion éthique sur l’expérimentation animale (CNREEA)*, under the agreement number C66-136-01. The CNREEA is part of the French Ministry of Higher Education, Research and Innovation.

### Cell lysis and chromatin shearing

Chromatin preparation was performed using the Diagenode ChIPmentation Kit for Histones, Cat. No. C01011009 and protocol with minor modifications. A total of 10,000 miracidia (1,000,000 cells based on the observation that one miracidium is composed of 100–120 cells) were resuspended in 1 mL 1x Hank’s balanced salt solution (HBSS), split into 2x500 µL and crushed with a plastic pestle in an Eppendorf tube on ice during ~1 min. For cross-linking, 13.5 µL of formaldehyde were added and tubes were incubated for 10 min at room temperature with occasional inversion. To stop cross-linking fixation, 57 µL of glycine were added and samples were incubated for 5 min at room temperature. Samples were centrifuged at 500xg, at 4°C, for 5 min. The pellet was resuspended in 2x1 mL of ice-cold Lysis Buffer iL1, combined and homogenized in a Dounce (pestle A) on ice for 5 min. After another centrifugation (500g, 5 min, 4°C), the supernatant was discarded and the pellet was resuspended in 1 mL of ice-cold Lysis Buffer IL2 and centrifuged (500xg, 5 min, 4°C). The supernatant was discarded and the pellet was resuspended in 100 µL of complete Shearing Buffer iS1 for each tube. Samples were sonicated with the Bioruptor Pico (Diagenode, Cat. No. B01080010) for 5 cycles (30 s ON and 30 s OFF). After transfer into new tubes, samples were centrifuged (16,000g, 10 min, 4°C). The supernatants were transferred into a new single tube (200 µl total) and 20 µl iS1 were added, yielding a total volume of 220 µl. The procedure was done in duplicate (named L and R in the following). Serial dilutions were done in iS1 to produce 100 µl equivalents of 10,000 miracidia (10 ^6^ cells), 1,000 miracidia (10 ^5^ cells), 100 miracidia (10 ^4^ cells), 50 miracidia (5,000 cells), 10 miracidia (10 ^3^ cells), five miracidia (500 cells) and one miracidium (100 cells) or 100 µl iS1 as negative control.

### Magnetic immunoprecipitation and tagmentation

Immunoprecipitation (IP) was performed on the Diagenode IP-Star Compact Automated System (Cat. No. B03000002) according to the ChIPmentation Kit for Histones User Guide and by following the manufacturer’s on-screen instructions. Antibody (Ab) coating time was set to 3 h, IP reaction to 13 h, washes to 10 min, and tagmentation to 5 min. For each sample the Ab coating mix was done with 4 µl anti-H3K4me3 (Diagenode, Cat. No. C15410003; mixture of lot A1051D and A1052D; raised in rabbit).

Stripping, end repair and reverse cross-linking were done as indicated in the User Guide.

### Input library tagmentation

In the ChIPmentation Kit for Histones (Diagenode, Cat. No. C01011009) the suggested strategy is to sequence one immunoprecipitated sample with a control immunoglobulin G (IgG) and to use it for sequencing normalization instead of the traditional input, which cannot be treated in exactly the same way as the immunoprecipitated samples. However, IgG are negative control samples, and the generation of such samples in low-amount approaches involves in our experience the use of a high number of amplification cycles that can induce some biases. A protocol for the tagmentation of the input sample was therefore set-up as follows.

For each immunoprecipitated sample, 1µL of sheared chromatin was kept aside before IP in the IP-Star. 1 µL of MgCl _2_ (Diagenode ChIPmentation kit for Histones, Cat. No. C01011009), 8 µL of molecular biology grade water, 10 µL 2xTagment DNA buffer and 1 µL of 100-fold in molecular-grade water diluted DNA tagmentation enzyme (Illumina 20034197, lot 20464427) were added to each 1 µL input. The tagmentation reaction was performed in a thermocycler for five minutes at 55°C. Then, 25 µL of 2xPCR NEB master mix (New England Biolabs M0541L, lot 10067165) was added to each input. The end-repair and de-cross-link were performed in a thermocycler for five minutes at 72°C followed by 10 minutes at 95°C. An aliquot of 2 µL was taken from each input and added to 8 µL of quantification mix. For each reaction, this quantification mix was composed of 0.3 µL of forward and reverse ATAC-seq primers (25 µM) ( Table 1, ( Buenrostro *et al.,* 2015), 1 µL SYBR Green 10X (Diagenode kit), 1.3 µL of molecular biology grade water and 5 µL of 2xPCR NEB master mix. While not formally tested, leftover primers of the ChIPmentation kit could probably also be used, but the volume must be adjusted as the primer pairs in the kit were 10 µM instead of 25 µM. Library amplification, purification, quality checking and sequencing steps were performed as for the immunoprecipitated samples (see below). 

**Table 1.** ATAC-seq primer indexes and sequences ( Buenrostro *et al.,* 2015).

Index ID	Sequence
Ad1_noMX:	AATGATACGGCGACCACCGAGATCTACACTCGTCGGCAGCGTCAGATGTG
Ad2.1_TAAGGCGA	CAAGCAGAAGACGGCATACGAGATTCGCCTTAGTCTCGTGGGCTCGGAGATGT
Ad2.2_CGTACTAG	CAAGCAGAAGACGGCATACGAGATCTAGTACGGTCTCGTGGGCTCGGAGATGT
Ad2.3_AGGCAGAA	CAAGCAGAAGACGGCATACGAGATTTCTGCCTGTCTCGTGGGCTCGGAGATGT
Ad2.4_TCCTGAGC	CAAGCAGAAGACGGCATACGAGATGCTCAGGAGTCTCGTGGGCTCGGAGATGT
Ad2.5_GGACTCCT	CAAGCAGAAGACGGCATACGAGATAGGAGTCCGTCTCGTGGGCTCGGAGATGT
Ad2.6_TAGGCATG	CAAGCAGAAGACGGCATACGAGATCATGCCTAGTCTCGTGGGCTCGGAGATGT
Ad2.7_CTCTCTAC	CAAGCAGAAGACGGCATACGAGATGTAGAGAGGTCTCGTGGGCTCGGAGATGT
Ad2.8_CAGAGAGG	CAAGCAGAAGACGGCATACGAGATCCTCTCTGGTCTCGTGGGCTCGGAGATGT
Ad2.9_GCTACGCT	CAAGCAGAAGACGGCATACGAGATAGCGTAGCGTCTCGTGGGCTCGGAGATGT
Ad2.10_CGAGGCTG	CAAGCAGAAGACGGCATACGAGATCAGCCTCGGTCTCGTGGGCTCGGAGATGT
Ad2.11_AAGAGGCA	CAAGCAGAAGACGGCATACGAGATTGCCTCTTGTCTCGTGGGCTCGGAGATGT
Ad2.12_GTAGAGGA	CAAGCAGAAGACGGCATACGAGATTCCTCTACGTCTCGTGGGCTCGGAGATGT
Ad2.13_GTCGTGAT	CAAGCAGAAGACGGCATACGAGATATCACGACGTCTCGTGGGCTCGGAGATGT
Ad2.14_ACCACTGT	CAAGCAGAAGACGGCATACGAGATACAGTGGTGTCTCGTGGGCTCGGAGATGT
Ad2.15_TGGATCTG	CAAGCAGAAGACGGCATACGAGATCAGATCCAGTCTCGTGGGCTCGGAGATGT
Ad2.16_CCGTTTGT	CAAGCAGAAGACGGCATACGAGATACAAACGGGTCTCGTGGGCTCGGAGATGT
Ad2.17_TGCTGGGT	CAAGCAGAAGACGGCATACGAGATACCCAGCAGTCTCGTGGGCTCGGAGATGT
Ad2.18_GAGGGGTT	CAAGCAGAAGACGGCATACGAGATAACCCCTCGTCTCGTGGGCTCGGAGATGT
Ad2.19_AGGTTGGG	CAAGCAGAAGACGGCATACGAGATCCCAACCTGTCTCGTGGGCTCGGAGATGT
Ad2.20_GTGTGGTG	CAAGCAGAAGACGGCATACGAGATCACCACACGTCTCGTGGGCTCGGAGATGT
Ad2.21_TGGGTTTC	CAAGCAGAAGACGGCATACGAGATGAAACCCAGTCTCGTGGGCTCGGAGATGT
Ad2.22_TGGTCACA	CAAGCAGAAGACGGCATACGAGATTGTGACCAGTCTCGTGGGCTCGGAGATGT
Ad2.23_TTGACCCT	CAAGCAGAAGACGGCATACGAGATAGGGTCAAGTCTCGTGGGCTCGGAGATGT
Ad2.24_CCACTCCT	CAAGCAGAAGACGGCATACGAGATAGGAGTGGGTCTCGTGGGCTCGGAGATGT

### Library amplification

To determine optimal number of library amplification cycles we proceeded as described in steps 5.1 to 5.5 of the ChIPmentation User Guide with modifications detailed below. We determined the number of amplification cycles for each library by using the number of cycles that corresponded to 1/3 of the qPCR amplification curve slope during the exponential phase. Results are shown in Table 6.

Amplification was done in step 5.7. After PCR, 48 µl of library amplification mix were AMPure-purified on the IP-Star using 86 µl of AMPure beads (1.8x). Instead of a resuspension buffer, we used ChIP grade water for a final volume of 20 µl.

### Library check and sequencing

Library fragment size and concentration was checked on an Agilent 2100 Bioanalyzer with a High Sensitivity DNA Assay v1.03. Paired-end sequencing (2x75 cycles) was performed at the Bio-Environnement platform (University of Perpignan, France) on a NextSeq 550 instrument (Illumina, USA).

### Bioinformatics analysis

Reads were quality-checked with FastQC, Galaxy version 0.72 and Galaxy 1 ( Smith, 2019). Adapters were detected in less than 4% of reads. Reads were aligned with the *S. mansoni* v7 reference genomes using Bowtie2 Galaxy version 2.3.4.3 ( Langmead & Salzberg, 2012)., the default sensitive settings.

Uniquely aligned reads were filtered from the BAM files using the XS: tag of Bowtie2, Galaxy version 2.3.4.3 and Galaxy 0 ( Langmead & Salzberg, 2012). PCR duplicates were removed with samtools rmdup Galaxy version 2.0.1 ( Li *et al.,* 2009). BAM files were subsampled to 3.8 M uniquely aligned reads with Picard DownsampleSam Galaxy version 2.18.2.1 (Broad Institute 2022, February 23) . Peakcalling was done with Peakranger (v1.17) Galaxy version 1.0.0 ( Feng *et al.,* 2011), with P value cut-off: 0.0001, false discovery rate (FDR) cut-off: 0.05, Read extension length 100 - 2,000 bp, Smoothing bandwidth: 99. Delta: 0.8, Detection mode: region. ChromstaR, Galaxy version 0.99.0 ( Taudt *et al.,* 2016) was used with a bin size same as Peakranger extension lengths and a step size of half a bin size. Miracidia genomic DNA libraries served as input. In ChromstaR postprocessing, the maximum posterior probabilities to adjust sensitivity of peak detection was set to 0.999. This kept broad peaks intact. We reasoned that gaps between peaks that were larger than a nucleosome are not biologically meaningful and peaks were merged with BEDTOOLS, Galaxy version 2.29.2 ( Quinlan & Hall, 2010) when they were ≤150 bp apart (the average length of DNA in a nucleosome).

Enrichment plots over metagenes were produced over 5,073 genes on the positive strand based on the canonical annotation v7 of *S. mansoni*.

## Results

### ChIPmentation has a sensitivity that is comparable to N-ChIP

**Figure 2.**
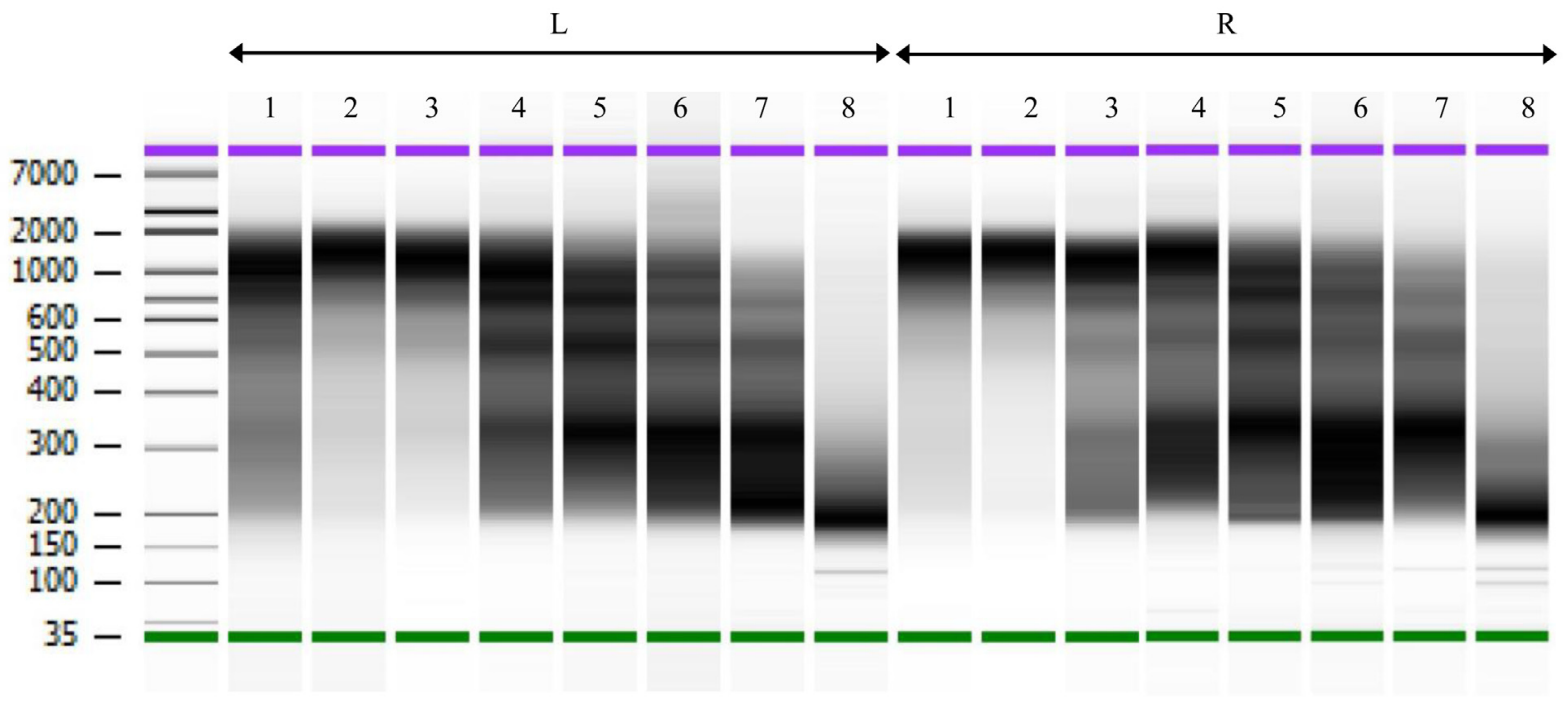
ChIPmentation library profiles for two technical replicates (L and R). Electrophoresis performed on BioAnalyser. Lanes 1–8 from left to right: 10 ^6^, 10 ^5^, 10 ^4^, 5x10 ^3^, 10 ^3^, 500, 100, and 0 cells. Libraries used in lanes L1, L3, L5 and L7, and R1, R3, R5 and R7 were subsequently sequenced. Size indicated on the left in base pairs.

We used *S. mansoni* miracidia which are composed of 100–120 cells as starting material, allowing for a good estimate of cell numbers. After the chromatin fragmentation and library amplification, 10 ^6^ to 10 ^4^ cell equivalents gave comparable Bioanalyzer profiles with peaks around 1kb. For 5,000 cells equivalents and below, fragments of smaller size became clearly visible. No high-molecular fragments were observed in the negative control without chromatin ( Figure 2 and Table 2).

**Table 2.** Cell number equivalents and library amplification cycles for ChIPmentation.

Position	Equivalent Miracidia	Equivalent cells	Amplification cycles L1-8	Amplification cycles R1-8	Sequenced
1	10,000	1,000,000	17	18	y
2	1,000	100,000	17	17	
3	100	10,000	18	19	y
4	50	5,000	19	18	
5	10	1,000	19	20	y
6	5	500	22	20	
7	1	100	22	22	y
8	0	0	26	25	

Alignments to the genome gave expected results (~50% uniquely aligned reads) for 10 ^6^ to 10 ^4^ cell equivalents, but dropped to ~35% with 10 ^3^ cells equivalent and were <20% for 100 cells equivalent. No contaminating DNA was detected ( Table 3).

**Table 3.** General statistics on ChIPmentation libraries. All libraries were downsampled to 3.8 M uniquely aligned reads.

Pos	Equivalent miracidia	Equivalent cells	Amplification cycles	qBit DNA HS ng/µl	Read pairs	Uniquely aligned	After deduplication	% unique non-dup	% to keep for 3.8 M
L1	10,000	1,000,000	17	23.2	5,728,173	7,969,894	6,775,525	0	0
R1	10,000	1,000,000	18	21.2	8,388,53	13,469,770	10,007,402	0	0
L3	100	10,000	18	25.2	12,537,243	19,157,585	14,417,614	0	0
R3	100	10,000	19	30.4	8,665,837	13,838,684	8,409,793	0	0
L5	10	1,000	19	13.8	11,436,716	17,920,411	8,664,467	0	0
R5	10	1,000	20	19.3	11,109,380	17,854,360	7,214,153	0	0
L7	1	100	22	22.0	11,662,120	15,737,202	4,095,477	0	0
R7	1	100	22	11.9	10,566,906	15,221,913	3,827,034	0	0

In order to compare ChIPmentation results to N-ChIP we re-analysed earlier data obtained by N-ChIP ( Augusto *et al.,* 2019) using the same data-cleaning, alignment and peak-calling parameters as for ChIPmentation ( Table 4). For ChIPmentation, peakcalling with Peakranger was highly robust for 10 ^6^ cells and delivered the expected values (based on earlier N-ChIP results). Below this cell equivalent, peak-calling became dependent on bin size ( Table 5).

**Table 4.** General statistics on N-ChIP libraries. When possible, libraries were downsampled to 3.8 M uniquely aligned reads.

Pos	Equivalent Miracidia	Equivalent cells	Amplification cycles	Read pairs	Uniquely aligned	After deduplication	% unique non-dup	% downsampled
A	8,000	800,000	14	13,129,021	11,299,195	10,763,378	41 %	35 %
B	100	10,000	14	21,415,343	5,409,960	2,477,749	6 %	100 %
C	10	1,000	14	29,857,139	12,170,093	6,718,570	11 %	57 %

**Table 5.** Optimization of peakcalling with Peakranger. Number of peaks identified for each condition. ChIPmentation on top. In bold: 1000 bp was selected as the best extension length and applied to N-ChIP data below. N-ChIP A is for 0.8x10 ^6^ cells.

cell equivalents	1,000,000			10,000			1,000			100		
	ChIPmentation			ChIPmentation			ChIPmentation			ChIPmentation	
Peakranger read extension length in bp	L1	R1	N-ChIP A	L3	R3	N-ChIP B	L5	R5	N-ChIP C	L7	R7
100	6,871	6,767		625	424		200	370		4,732	5,683
300	10,369	9,545		2,125	1,211		499	635		3,944	5,264
600	10,581	9,924		3,513	2,823		610	813		3,197	3,816
**1,000**	**9,632**	**9,835**	**8,320**	**4,033**	**2,749**	**3,888**	**695**	**811**	**131**	**3,098**	**3,416**
1,500	9,901	9,426		3,315	2,925		847	837		4,019	3,526
2,000	9,067	8,353		3,052	2,631		626	693		4,336	3,897

We iteratively identified 1,000 bp as best-read extension length. Using the HMM-based ChromstaR improved peak calling for ChIPmentation 10 ^4^ cell equivalent, but not for 10 ^3^ or 100 cells. We obtained comparable results for ChIPmentation of 10 ^6^, 10 ^4^ cells and N-ChIP ~10 ^6^, 10 ^4^ and 10 ^3^ cells ( Table 6).

**Table 6.** Peakcalling with HMM-based ChromstaR before and after merging adjacent peaks (in bold).

Cell equivalents	1,000,000		10,000		1,000		100	
ChromstaR bin 1000, step 500, post prob 0.999	All	merged	All	Merged	All	Merged	All	Merged
ChIPmentation	13,565	**6,682**	7,194	**5,504**	3,137	**2,876**	10,665	**10,522**
N-ChIP	6,262	**6,186**	7,058	**6,922**	5,296	**5,129**		

**Figure 3.**
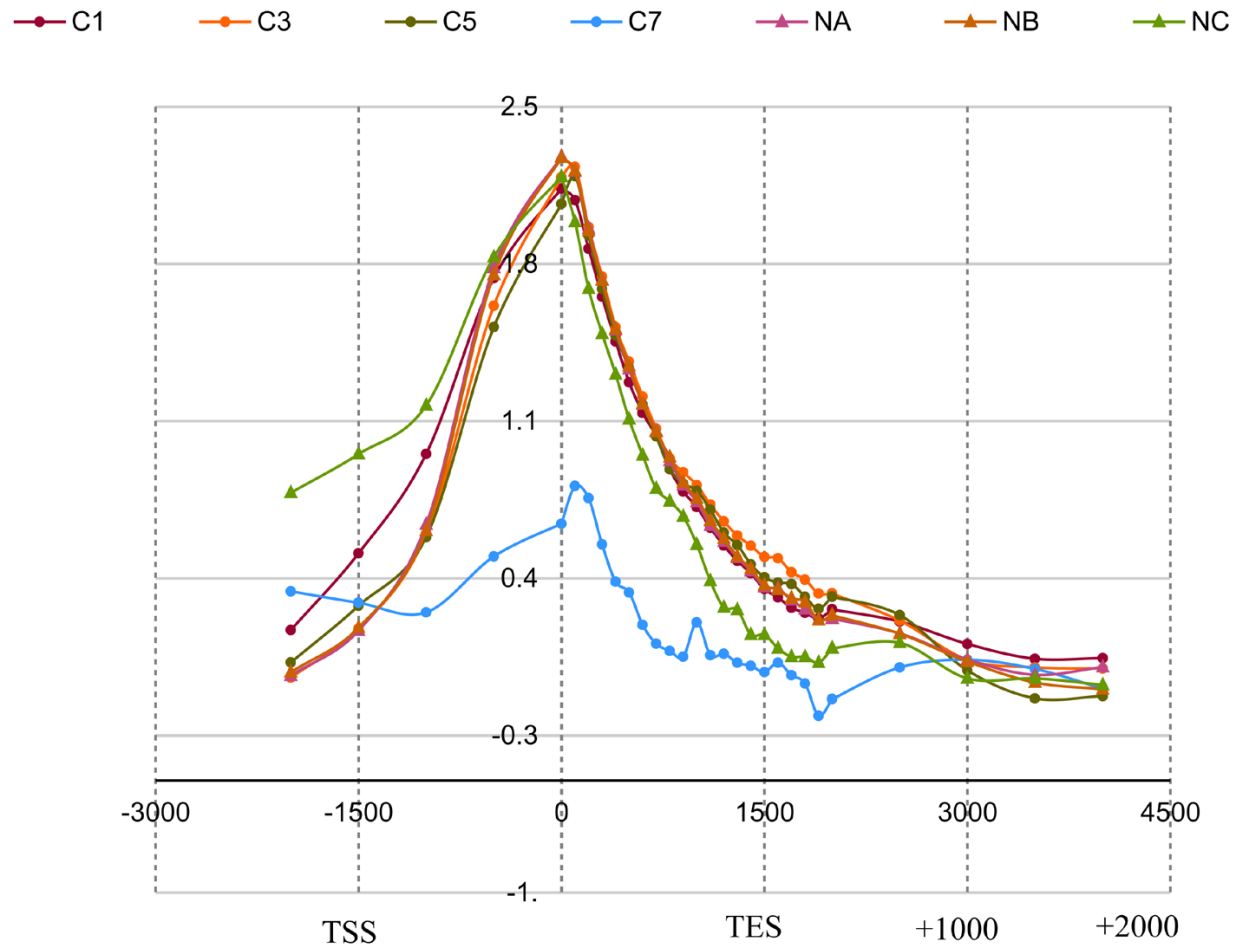
Average metagene profiles over 5,073 plus strand genes for ChIPmentation and N-ChIP. ChIPmentation samples were 10 ^6^ (C1), 10 ^4^ (C3), 10 ^3^ (C5) and 100 cell equivalents (C7). N-ChIP samples were 0.8x10 ^6^ cells (NA), 10 ^4^ (NB) and 10 ^3^ (NC). X-axis: bp upstream, within and downstream of genes. TSS/TES for transcription start and end sites. Y-axis: log(observed/expected). Not all genes contributed to the profiles as only roughly half of the genes show a H3K4me3 peak at the TSS.

ChromstaR metagene profiles showed consistent profiles for ChIPmentation 10 ^6^, 10 ^4^ and 10 ^3 ^cells, and all N-ChIP, but not for ChIPmentation on 100 cell equivalents ( Figure 3).

**Figure 4.**
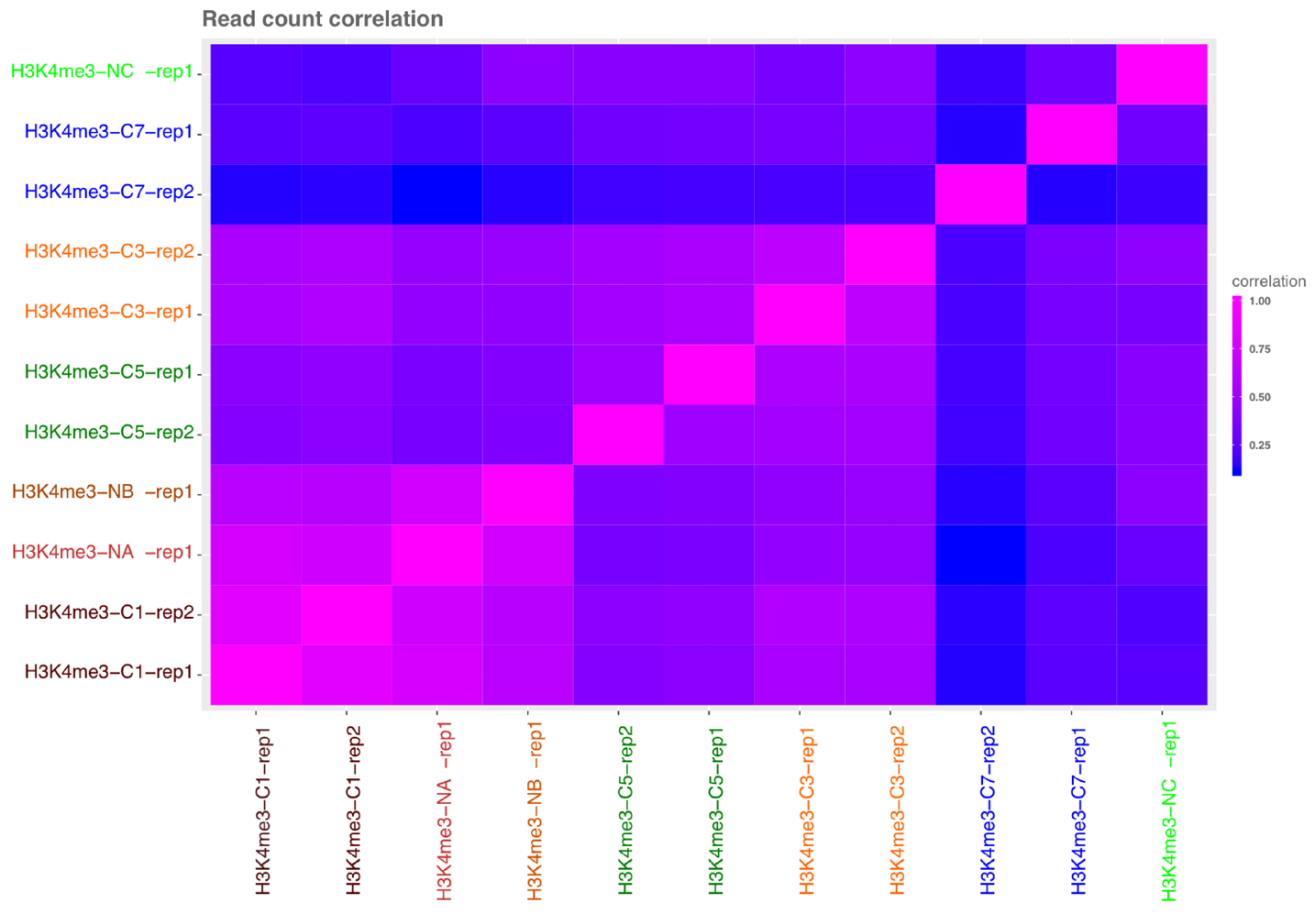
ChromstaR read count correlations between libraries (lowest 0, highest 1). L and R samples were considered as replicates 1 and 2 for ChromstaR analysis. ChIPmentation 10 ^6^ (C1), 10 ^4^ (C3), 10 ^3^ (C5) and 100 cell equivalent (C7). N-ChIP 0.8x10 ^6^ cells (NA), 10 ^4^ (NB) and 10 ^3^ (NC).

ChromstaR allows for estimating the correlation of chromatin profiles based on read counts ( Figure 4), and indicates high correlations between ChIPmentation 10 ^6^, and N-ChIP ~10 ^6^, 10 ^4^.

**Figure 5.**
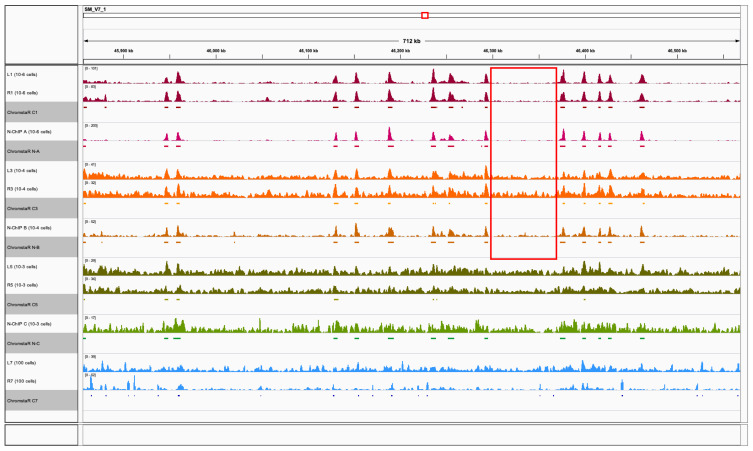
Genome browser screen shot of a typical region of the *S. mansoni* genome with Peakranger chromatin profiles for visual inspection and HMM model-based ChromstaR peak regions (grey underlay). ChIPmentation 10 ^6^ (L1, R1), 10 ^4^ (L3, R3), 10 ^3^ (L5, R5) and 100 cell equivalent (L7, R7), and N-ChIP 0.8x10 ^6^ cells (N-A), 10 ^4^ (N-B) and 10 ^3^ (N-C). Color codes as in previous figures : ChIPmentation 10 ^6^ (L1, R1) in dark red, N-ChIP 0.8x10 ^6^ cells (N-A) in magenta, ChIPmentation 10 ^4^ (L3, R3) in orange, N-ChIP 10 ^4^ (N-B) in dark orange, ChIPmentation 10 ^3^ (L5, R5) in green, N-ChIP 10 ^3^ (N-C) in light green and ChIPmentation 100 cell equivalent (L7, R7) in light blue The region circled in red illustrates higher background for 10 ^4^ cells equivalent in ChIPmentation C3 (orange, replicates L3, R3) than in N-ChIP B (dark orange).

All other overall chromatin profiles were below 0.75 correlation coefficient. This is surprising given the high similarity of metagene profiles. Visual inspection of peaks and profiles showed that peaks were actually correctly identified by ChromstaR (but much less by Peakranger) in ChIPmentation until 10 ^4^ cells, but there was higher background than in N-ChIP which probably decreased the correlation for lower cell equivalents ( Figure 5).

BEDTools intersect with default parameters identified 5,948 common peaks between the 6,682 ChIPmentation peaks and the 6,186 N-ChIP peaks obtained with 10 ^6^ cells. This corresponds to an empirical FDR of 0.89–0.96%. This FDR cannot distinguish between biological and technical variation.

### ChIPmentation input library can rapidly be produced in parallel to the automated procedure

**Figure 6.**
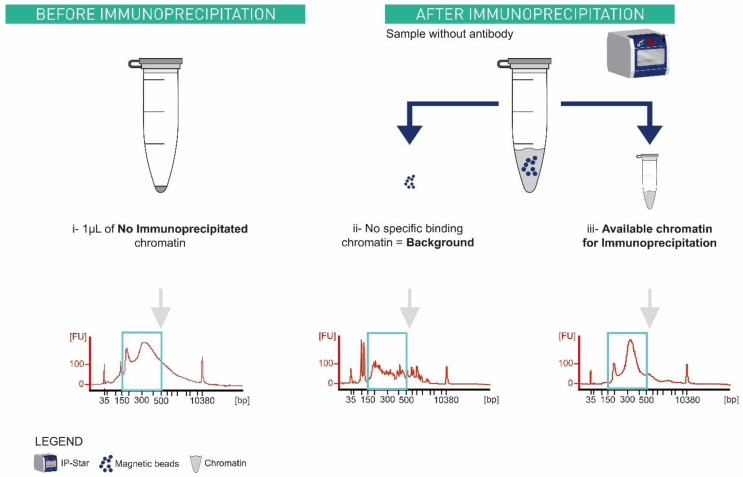
Types of inputs generated during ChIPmentation and their associated bioanalyzer profiles. X axis represents the size in base pair, and Y axis represents the fluorescence intensity. Ideal library size is between 150 bp and 500 bp (blue rectangle). (i) 1µL aliquot of one sample chromatin taken before its immunoprecipitation. (ii) and (iii) are from a supplementary sample where immunoprecipitation in the IP-Star is done without antibody. After immunoprecipitation, this supplementary sample contains magnetic beads with the (ii) non-specifically binding chromatin and the supernatant which is the (iii) available chromatin for immunoprecipitation. (ii) No specific binding chromatin which did not deliver a usable library.

After having formally established that automated ChIPmentation had a comparable sensitivity to our routine N-ChIP procedure, we aimed to identify the optimal way to produce input libraries for control of unspecific enrichment. The production of input chromatin is “built-in” the N-ChIP protocol ( Cosseau *et al.,* 2009; de Carvalho Augusto *et al.,* 2020; Roquis *et al.,* 2018) and needed to be adapted to the automated ChIPmentation procedure. During a ChIPmentation experiment three types of input can be considered ( Figure 6): (i) 1 µL of chromatin before immunoprecipitation, (ii) chromatin that binds non-specifically to any support, and (iii) chromatin truly available for IP. In (i), an aliquot of 1 µL is taken from the sample before *immunoprecipitation*. The two other types (input library ii and iii) need a supplementary sample in which mock IP is done without antibody. After IP, this supplementary sample contains magnetic beads with the non-specifically bound chromatin and the supernatant, which is the available chromatin for IP.

Only (i), *i.e.* before IP chromatin and (iii) *i.e.* free chromatin available for IP give ideal library sizes ( Figure 6). We decided to optimize the input protocol for option (i), non-immunoprecipitated chromatin because it does not occupy a slot in the IP-Star. In addition, during the preliminary test, 1 µL chromatin input showed a lower Ct compared to option (iii) input, which means that it requires fewer amplification cycles (data on Zenodo).

**Figure 7.**
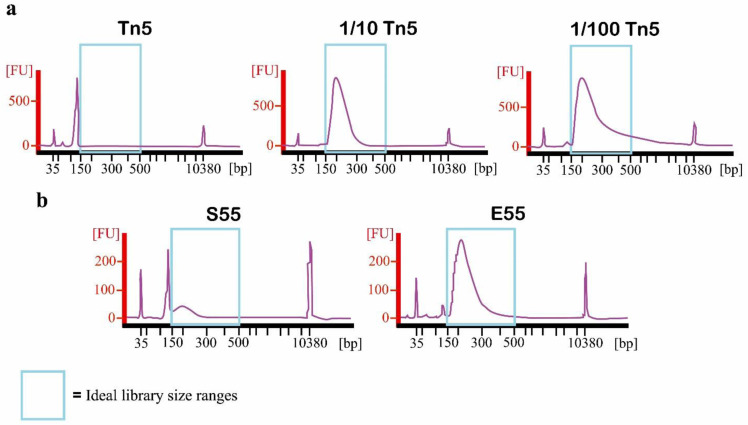
Bioanalyzer profiles for input libraries performed with different Tn5 and SDS conditions. **a)** with undiluted Tn5 ( **Tn5**), 10-fold diluted Tn5 ( **1/10 Tn5**) and 100-fold diluted Tn5 ( **1/100 Tn5**). **b)** 10-fold diluted Tn5 with 0.2% SDS ( **S55**) and 10-fold diluted Tn5 without 0.2% SDS ( **E55**). X axis represents the size in base pair, and Y axis represents the fluorescence intensity. Ideal library size is between 150 bp and 500 bp (blue rectangle).

For the preparation of the 1 µL chromatin input libraries, we identified two critical parameters. The first one was the dilution of the tagmentation enzyme. Using undiluted Tn5 caused complete over-tagmentation. Between 10- and 100-fold dilutions in water delivered optimal results ( Figure 7A).

**Figure 8.**
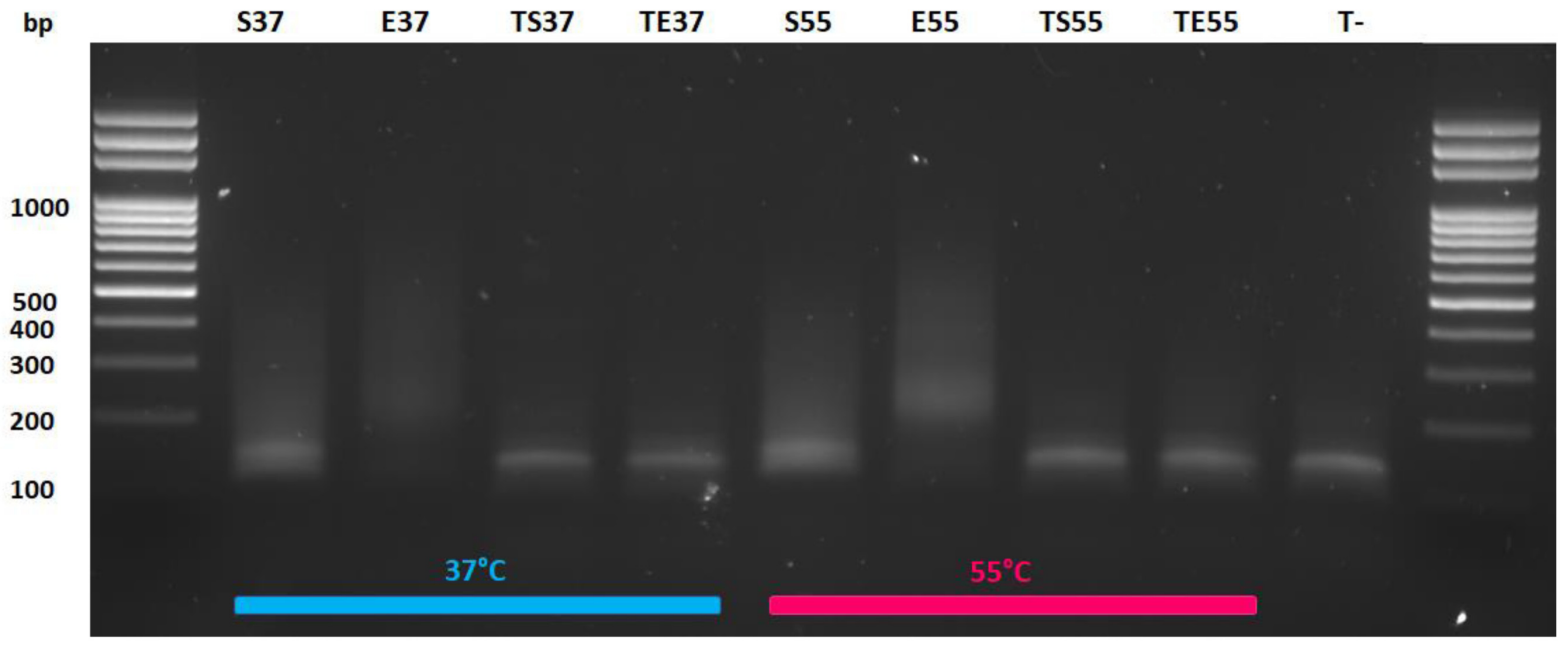
Picture of size separation of PCR products after qPCR for input library quantification by electrophoresis. Electrophoresis was performed through an agarose gel stained with ethidium bromide. Tagmentation was performed at 37°C and 55°C with 10-fold diluted Tn5. Sample legend: ( **S**) SDS, ( **E**) No SDS, ( **TS**) no Tn5 enzyme with 0.2% SDS, ( **TE**) no Tn5 enzyme and no SDS, ( **T-**) qPCR negative control without chromatin. Ideal library size is between 150 bp and 500 bp. Only **E** samples had the right library size.

Secondly, we found that, in our hands, there was no need to add tagmentation neutralizer (0.2% sodium dodecyl sulfate [SDS]) after tagmentation ( Figure 7-B and Figure 8). This actually inhibits the PCR amplification step ( Picelli *et al.,* 2014). Interestingly, parameters like tagmentation temperature (37°C-55°C) ( Figure 8), tagmentation time (2–10 min) and addition of MgCl _2_ did not have a critical effect on input library generation.

## Discussion

As many methods, ChIPmentation had been developed using readily available but highly artificial cell cultures ( Schmidl *et al.,* 2015). The transition from such model systems to non-model species and ecologically realistic conditions is sometimes very difficult or even impossible. Here we showed that the automated ChIPmentation can be done with a parasitic flatworm and delivers results comparable to N-ChIP, the current method of choice for this species. However, the method is roughly two times faster and requires roughly six times less hands-on time. Since the procedure is done on a pipetting robot with on-screen instructions for the researcher, in our experience, training time was reduced to about a week.

We empirically define the limit for the robust detection of peaks with ChIPmentation to be 100,000 cells equivalent per antibody reaction, with 10,000 being the absolute limit if background is acceptable. Operational limit for N-ChIP is 10,000 cells equivalent, confirming our previous results. To avoid variations that might be introduced by small errors in the estimation of cell numbers, we arbitrarily doubled this lower cell limit and established ≥20,000 cells as the lower limit for the routine ChIPmentation-seq procedures in *S. mansoni*. This is a little higher than what has been described in Human cells where good results have been obtained with as little as 5,000 cells.

To improve signal to noise ratio and reduce background in ChIPmentation, it could be useful to increase the washing time (currently 10 min) and speed (currently medium) but we recommend increasing cell number rather than to invest in washing optimization.

Using 1 µL of non-immunoprecipitated chromatin for the reference input library production is the best compromise to save space in the IP-Star, experiment time and biological materials when one is restricted by quantity.

This ChIPmentation protocol is not limited to miracidia cells. We also performed this protocol on *S. mansoni* adult worms and sporocysts. It should be noted that the number of sonication cycles needs to be experimentally determined and adapted for each sample type before proceeding to ChIPmentation experiments.

A new version of the ChIPmentation solution, called µChIPmentation for Histones (Diagenode, Cat. No. C01011011), has also been released recently in order to improve the quality for low-amount samples. This relies on a reduced number of steps, especially during chromatin preparation, and reduced number of tube transfers, in order to avoid DNA loss. It also contains a new protocol to process the non-immunoprecipitated chromatin input samples up to the sequencing step. This new version of µChIPmentation may be a good alternative for experiments on very low cell numbers in the future.

## Data availability

### Underlying data

NCBI SRA: Automated ChIPmentation procedure on limited biological material of the human blood fluke Schistosoma mansoni. Accession number: PRJNA816041, https://identifiers.org/ncbiprotein:PRJNA816041

This project contains the Fastq data of the ChIPmentation libraries.

Zenodo: Supporting information for "Automated ChIPmentation procedure on limited biological material of the human blood fluke Schistosoma mansoni", https://doi.org/10.5281/zenodo.6375548 ( Grunau, 2022)

This project contains the following underlying data:

- 20200612-1_Report.pdf (qPCR report for inputs 1µL and available chromatin ("Row7"), see Figure 6)
- 20200919-1_Report.pdf (qPCR report for testing SDS after Tn5, see Figure 7B and Figure 8)
- 20200921-1_Report.pdf (qPCR report for comparing inputs with enzyme of Diagenode kit and our protocol with other enzyme Tn5)
- CG_Ro_1_High Sensitivity DNA Assay_DE13805677_2019-06-20_09-10-48.pdf (DNA assay file underlying Figure 2)
- CG_Ro_2_High Sensitivity DNA Assay_DE13805677_2019-06-20_10-12-12.pdf (DNA assay file underlying Figure 2)
- gel qpcr test input sds_01.Tif

Data are available under the terms of the Creative Commons Attribution 4.0 International license (CC-BY 4.0).
