# Supplementary material for: Automated ChIPmentation procedure on limited biological material of the human blood fluke Schistosoma mansoni
Source: Wellcome Open Res. 2024 Feb 13;7:133. Originally published 2022 Apr 11. [Version 2] doi: 10.12688/wellcomeopenres.17779.2 (PMC10372461; doi:10.12688/wellcomeopenres.17779.2)
Supplement: Peer review history

# Reviewer report: Reviewer response for version 1

**DOI:** 10.21956/wellcomeopenres.19678.r61954

**Published:** 2023-07-26

**Authors:** Qijun Chen

**Affiliations:** 1 Shenyang Agricultural University, Liao Ning Sheng, China

The methodology described in the manuscript represents a great improvement to the ChIP-Seq related studies in the interest of very limited biological materials. Many parasite species are not be able to proliferate outside their hosts, the approach presented here will improve studies in the parasitology field, though not limited. I think the protocol developed by the group is very interesting, thus is suggested for expansion in investigation on other organisms. The manuscript is recommended for indexing with minor revisions.

1. It would be more convincing if the experiments of the ChIPmentation were performed in parallel with the “native ChIP-Seq”, as described in Fig 1. Here the authors only referred previous data which were not generated with the same batches of materials.
2. The authors need to discuss the robustness or efficiency of the transposases, and the sources of the enzymes.

Is the rationale for developing the new method (or application) clearly explained?

Yes

Is the description of the method technically sound?

Yes

Are the conclusions about the method and its performance adequately supported by the findings presented in the article?

Yes

If any results are presented, are all the source data underlying the results available to ensure full reproducibility?

Yes

Are sufficient details provided to allow replication of the method development and its use by others?

Yes

Reviewer Expertise:

Molecular parasitology

I confirm that I have read this submission and believe that I have an appropriate level of expertise to confirm that it is of an acceptable scientific standard.

# Reviewer report: Reviewer response for version 1

**DOI:** 10.21956/wellcomeopenres.19678.r61955

**Published:** 2023-07-20

**Authors:** Renata Guerra-Sá

**Affiliations:** 1 Universidade Federal de Ouro Preto, Ouro Preto, State of Minas Gerais, Brazil

The data presented by the group are very interesting, especially when applied to the human parasite *Schistosoma mansoni*, which, in addition to not being a model organism, does not have a genome considered finalized. Thus, the methodology described in this paper opens up an interesting perspective for the study of the different evolutionary forms of the parasite, which will certainly provide interesting epigenetic mechanisms, especially with regard to the host-parasite interaction. I have only two considerations, minor review:

- I understand that two steps are critical to success in the CHIP methodology. The sonication conditions and DNA loss between each chromatin purification step. Thus, I think that these points could be better explored in the discussion and;
- I missed a more detailed comment in terms of cost and number of biological replicas and the size of libraries when comparing ChIPmentation and N-ChIP techniques.

Is the rationale for developing the new method (or application) clearly explained?

Yes

Is the description of the method technically sound?

Yes

Are the conclusions about the method and its performance adequately supported by the findings presented in the article?

Yes

If any results are presented, are all the source data underlying the results available to ensure full reproducibility?

Yes

Are sufficient details provided to allow replication of the method development and its use by others?

Yes

Reviewer Expertise:

Molecular Biology and Epigenetics

I confirm that I have read this submission and believe that I have an appropriate level of expertise to confirm that it is of an acceptable scientific standard.

# Reviewer report: Reviewer response for version 1

**DOI:** 10.21956/wellcomeopenres.19678.r50460

**Published:** 2022-05-24

**Authors:** Nolwenn M. Dheilly

**Affiliations:** 1 UMR Virology ANSES/INRAE, ENVA, Animal health laboratory, 14 rue Pierre et Marie Curie, Maisons Alfort, France

The manuscript by Lasica et al. provides a description of the automated ChIPmentation procedure applied to a trematode parasite. The authors argue that the automated procedure is as sensitive as CHIP-Seq and define an operational limit of >20,000 cells. I think the publication of the method is of significance but the manuscript needs to be improved. I suggest a more thorough analysis of the data collected with improved statistics to give the results more strength.

-  I found the manuscript difficult to read for a number of reasons.
  1. Given that the authors compare two techniques, they should more explicitly discuss sensitivity and specificity of the methods. I understand there is not "reference" material but I suggest posing the hypothesis that CHIP-seq is right and use these result as a reference to quantifying explicitly the error rates with different quantities of input material.  Based on results from BEDTools interest, there are 734 peaks identified by CHIPmentation that have not been found by CHIPseq: are these false positives? There are 238 peaks found by CHIPseq not found by CHIPmentation: are these false negative? What are the differences in estimated false positives and false negatives when a different quantity of input material is used? This is particularly important to calculate and compare with 10,000 cells given the conclusions of the article.
  2. The figures should be revised to provide  more easy-to-read labels. For example, I had to go back and forth between the figure and legend to figure out what is C, C3 etc. Authors should also make sure that appropriate labels for axes are provided. (more detailed suggestions on each figure below). 
  3. Some informations provided in the tables are neither presented nor discussed. I do not know what they mean and what they can be used for. can this be described in the table legend?
  4. It can be difficult for some readers when the same result - size distribution of libraries - is presented in different formats: electrophoresis gel of Bioanalyzer in Fig 2, actual capillary RNA elextrophoresis plot from the Bioanalyzer in Fig 6 and 7, and then gel electrophoresis for fig 8.
-  I think the impact of the mansucript would be greatly increased if the authors provide statistical analyses: is there a significant difference between the number of peaks called with ChIPmentation and N-CHIP ? What is the percentage of peaks from one experiment also present in another? 
- I am not convinced by the >20,000 cells threshold suggested by the authors. Results presented in Fig 4 and 5 rather show that there is high background noise at 10,000 cells and that more accurate results are obtained at 100,000 cells. The method has never been tested with 20,000 cells and there are no statistical or modelisation results to support this threshold. Can something be dones to better support this operational limit?

More minor and detailled comments:

- Methods: homogenize "500g" and "500xg"; "µL" and "µl".
- "20µl iS1 were added" replaced with "was".
- Figure 1: Image quality is too poor for publication. It was blurry.
- Input Library prep, second paragraph: a sentence can not start with a number, "1" must be spelled out to "One".
- Library amplification: Results are shown in table 6 or table 2?
- Results, paragraph 1: I have no idea how the text relates to table 2. I disagree with the statement that 10 ^6 ^and 10 ^4^ gave comparable results. In Figure 2, L1 appears different to R1 and L3 appears different to L3.
- Table 2: spell out “yes”.
- Table 3: alignment to the genome gave expected results (50%): maybe provide percentage in the table? Is there a problem with the number of read pairs for R1? Can you explicitely explain what is provided in each column in the table legend?
- Table 5: below 1000, peak calling became dependent on bin size: How did you determine this threshold? Why has this number of peaks not been calculated for N-CHIP with other read extension length?
- Figure 3: “consistent profiles” of metagene profiles except for 100 cells. Please revise the labels on the figure to make it easier to read. It seems that NC profile is also different from the others. Can you add the 95% confidence interval? Can you provide statistical analyses to test whether the profiles actually differ?
- Figure 4: What does ChromstaR does? Is this a genome wide correlation analysis? Is this spearman’s rank or pearson correlation? Can you please run statistical analyses on correlation results to help us determine whether the correlation results are significant?
- Figure 5: the “ central figure” from the Genome browser screen can be kept but the “labels” from the print screen need to be rewritten to make them easier to read, including the sequence length etc. The figure could be further described in the text to explain what else can be seen outside the box.
- Figure 7: how many replicates were run? Are 1/10 Tn5 and E55 the same?
- Results of evaluation of tagmentation time (2-10 min) and addition of MgCL2 have not been provided.
- Figure 8: it is very difficult to see the bands. Can you play with the figure contrast / light ? Why not using the Bioanalyzer here? The “no Tn5” results have not been discussed in the text.

Is the rationale for developing the new method (or application) clearly explained?

Yes

Is the description of the method technically sound?

Yes

Are the conclusions about the method and its performance adequately supported by the findings presented in the article?

Partly

If any results are presented, are all the source data underlying the results available to ensure full reproducibility?

Yes

Are sufficient details provided to allow replication of the method development and its use by others?

Yes

Reviewer Expertise:

Parasitology, Immunology, functional genomics, virology

I confirm that I have read this submission and believe that I have an appropriate level of expertise to confirm that it is of an acceptable scientific standard, however I have significant reservations, as outlined above.
